# A Modified Implementation Mapping Methodology for Evaluating and Learning From Existing Implementation

**DOI:** 10.3389/fpubh.2022.836552

**Published:** 2022-03-23

**Authors:** Marie-Therese Schultes, Bianca Albers, Laura Caci, Emanuela Nyantakyi, Lauren Clack

**Affiliations:** ^1^Faculty of Medicine, Institute for Implementation Science in Health Care, University of Zurich, Zurich, Switzerland; ^2^Department of Infectious Diseases and Hospital Epidemiology, University Hospital Zurich, Zurich, Switzerland

**Keywords:** Implementation Mapping, implementation strategies, existing implementation, stakeholder engagement, implementation experience, tailored implementation

## Abstract

When empirically supported interventions are implemented in real-world practice settings, the process of how these interventions are implemented is highly relevant for their potential success. Implementation Mapping is a method that provides step-by-step guidance for systematically designing implementation processes that fit the respective intervention and context. It includes needs assessments among relevant stakeholders, the identification of implementation outcomes and determinants, the selection and design of appropriate implementation strategies, the production of implementation protocols and an implementation outcome evaluation. Implementation Mapping is generally conceptualized as a tool to prospectively guide implementation. However, many implementation efforts build on previous or ongoing implementation efforts, i.e., “existing implementation.” Learnings from existing implementation may offer insights critical to the success of further implementation activities. In this article, we present a modified Implementation Mapping methodology to be applied when evaluating existing implementation. We illustrate the methodology using the example of evaluating ongoing organized colorectal cancer screening programs in Switzerland. Through this example, we describe how we identify relevant stakeholders, implementation determinants and outcomes as well as currently employed implementation strategies. Moreover, we describe how we compare the types of strategies that are part of existing implementation efforts with those that implementation science would suggest as being suited to address identified implementation determinants. The results can be used for assessing the current state of implementation outcomes, refining ongoing implementation strategies, and informing future implementation efforts.

## Introduction

When implementing empirically supported interventions in real-world settings, planning implementation processes that comprise a good fit between implementation strategies, the respective intervention, and context is a challenging task. Implementation Mapping is an approach based on Intervention Mapping (1) that provides practical guidance and supports systematically planning implementation processes (2). As a participatory approach, it involves engaging intervention users and implementers in the respective setting. Implementation Mapping has been used for prospectively planning implementation in a variety of fields, such as cancer prevention and control (3) and chronic pain management (4). The process follows five steps: identifying stakeholders and conducting needs assessments, identifying implementation outcomes and determinants, designing implementation strategies, creating implementation protocols, and evaluating implementation outcomes (2).

Benefits of Implementation Mapping include a more transparent selection of implementation strategies that makes it easier to replicate selection processes in similar studies (3). Accordingly, reasons for choosing implementation strategies as well as these strategies' potential mechanisms of action are more explicitly documented (4), which is particularly helpful for presenting the results of the Implementation Mapping process to involved stakeholders (3). Working closely with and understanding the needs of stakeholders is another key element of Implementation Mapping (2). This approach provides practical and systematic guidance on how to do that and thus complements the description of implementation processes offered by implementation frameworks.

Originally, Implementation Mapping was conceptualized as a tool to prospectively guide future implementation actions. However, implementation processes often build on previous and ongoing implementation efforts. In this article, we discuss how to use Implementation Mapping for evaluating and learning from existing implementation to inform future implementation efforts. We define existing implementation as the entirety of processes and strategies that are currently or were previously employed in a system to implement an intervention. The strategies employed by existing implementation efforts may vary in the extent to which they are guided by practical expertise and/or current best evidence on quality implementation.

Evaluating existing implementation is especially relevant for interventions that have been part of a health system over long periods of time and for which implementation gaps have been identified. It is also relevant for interventions that have recently been introduced to practice, but for which resources were insufficient to conduct initial systematic implementation planning. When evaluating existing implementation, engaging stakeholders to build on their implementation knowledge and experience is highly important. Accordingly, the participatory approach that is central to Implementation Mapping is also central to this modified methodology.

### Existing Implementation

So far, there has been no common terminology for describing existing implementation efforts. Lau et al. (5) contrast “investigator-driven implementation” with “system-driven implementation.” Powell et al. (6) describe existing implementation processes as “implementation as usual” and emphasize a need for studies analyzing current implementation processes in relation to strategies that would be recommended by implementation science.

For describing previous or ongoing implementation efforts, we propose the term “existing implementation” since it points at implementation processes being targeted efforts by stakeholders in the system (5), although these might not be explicitly based on implementation science. For example, when evaluating existing implementation of empirically supported interventions in organizations specialized in autism spectrum disorders, Drahota et al. (7) found that agencies informally followed steps described in the EPIS framework (8), although a structured implementation was not reported.

Evaluating existing implementation can provide a useful overview of strategies that stakeholders already employ to implement interventions in their respective settings. For example, their feasibility, acceptability, or effectiveness can be assessed when planning refined implementation activities. At the same time, stakeholders' practical expertise that drives existing implementation can be harnessed to inform future implementation efforts. Moreover, building on existing implementation structures and processes when designing implementation strategies bears the potential of increased cost-efficiency. Descriptions of how to assess previous and ongoing implementation efforts are scarce. Here, Implementation Mapping can be used to systematically evaluate existing implementation efforts in a participatory process.

### Stakeholder Engagement

When evaluating existing implementation, it is crucial to consider the experience and expertise of involved stakeholders, including decision makers, adopters, and implementers “on the ground.” Although these stakeholders might not be experts in implementation science, they hold implementation expertise that relates to their respective role and setting. Accordingly, by working together with stakeholders, their practice setting expertise can be merged with the evaluation team's process expertise.

The relevance of engaging stakeholders to improve the design of processes is widely discussed in both implementation and evaluation research. For example, Ramanadhan et al. (9) highlight the benefits of stakeholder engagement in implementation research for an appropriate selection of interventions, developing effective recruitment and retention strategies and capacity building on the part of stakeholders and researchers. In evaluation studies, including stakeholders in decisions about design, desirable outcomes and measures leads to more positive attitudes toward the evaluation process and contributes to both a higher use of evaluation results and internal evaluation capacity building (10).

Identifying stakeholders for an Implementation Mapping process is most likely an iterative process that can include expert interviews, focus groups or snowball sampling (11). When assembling a group of stakeholders, their potential interests, influence, and support for or skepticism toward the intervention, implementation and evaluation process should be considered (11). In an interview study with stakeholders from different health system levels (12), the participants described engagement as starting early in the process, involving two-way communication and ranging from information sharing to shared decision-making. However, the processes and actions that stakeholder engagement entails have not been defined consistently in implementation science (12) and there is little practical guidance on how to include stakeholders' expertise in implementation processes (13).

## Implementation Mapping for Evaluation of Existing Implementation

**Figure 1:**
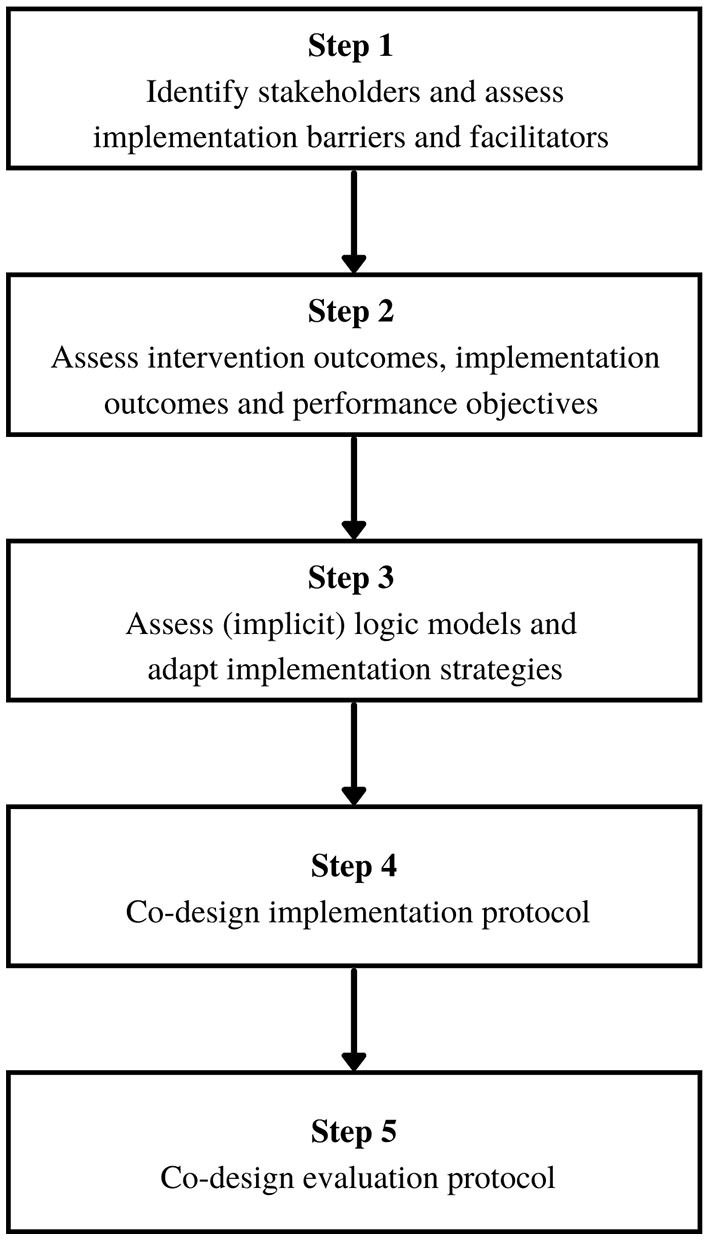
Implementation Mapping for evaluating existing implementation.

In the following, we present a roadmap for applying Implementation Mapping to the evaluation of existing implementation. In our description, we assume that an external evaluation team is assigned to evaluate existing implementation of a particular intervention and to improve the implementation process together with stakeholders. Figure 1 displays the steps of the adapted framework.

(1) *Identify stakeholders and assess implementation barriers and facilitators*:

The purpose of step 1 is to gain an overview of stakeholders' implementation experience with an intervention and their needs for continuing the implementation. The evaluation team identifies stakeholders who have been involved in the implementation process so far. Here, it is essential to identify intervention champions and formal as well as informal implementation leaders (14). For this purpose, a stakeholder mapping procedure may be helpful (15). Potentially, there is even an implementation team (16) or other entity that can guide change processes and function as a point of contact. Stakeholders' implementation experience is assessed with a focus on the barriers and facilitators that they have met at multiple levels of their service system. Preferably, this is done in workshops, focus groups or individual interviews and guided by an implementation determinants framework, such as the Consolidated Framework for Implementation Research (14).

(2) *Assess intervention outcomes, implementation outcomes, and performance objectives*:

The purpose of step 2 is to create clarity around the intended outcomes for an implementation process and the degree to which stakeholders have been able to achieve these. The evaluation team identifies and—if possible—assesses the intervention and implementation outcomes that stakeholders initially intended to pursue. Intervention outcomes are indicators for the effectiveness of the intervention and may have been formulated at the beginning of the existing implementation process. Implementation outcomes are indicators for the effectiveness of implementation strategies (17). Depending on the ongoing process, these may need to be made explicit in collaboration with stakeholders. Both types of outcomes are discussed with stakeholders to generate shared understanding about what has been accomplished so far and which barriers and facilitators influenced this accomplishment. It is also discussed whether the current range of intervention and implementation outcomes needs to be refined, considering the current state of the implementation process and the determinants that have been identified in step 1. Finally, it is crucial to define performance objectives, i.e., concrete tasks to be solved by implementers to achieve revised outcomes. This process is described in detail by Fernandez et al. (2).

(3) *Assess (implicit) logic models and adapt implementation strategies*:

The purpose of step 3 is to generate an overview of implementation strategies that are already in use and to understand the degree to which these could be adjusted to improve implementation. The evaluation team asks stakeholders, who have been involved in the existing process as implementation agents, to describe employed implementation strategies, i.e., “methods or techniques used to enhance the adoption, implementation, and sustainability of a clinical program or practice” (18). Here, reasons for choosing initial implementation strategies should be explored. For example, strategies may have been chosen due to available resources or opportunities, or they may be based on implicit or explicit theories of change. Most likely, implementers had implicit theories about how strategies would lead to certain outcomes. Making these theories explicit in logic models is helpful for prompting discussions about how employed strategies can lead to desired results. This necessitates the use of insights gained through step (1) and (2) and allows for rating already employed strategies in terms of their fit with previously identified implementation determinants. For this purpose, both the CFIR-ERIC matching tool (19) and Haley et al.'s (20) description of methods for tracking modifications to employed implementation strategies can be helpful resources. As a result of these discussions, employed implementation strategies may be adapted, discontinued, expanded, or replaced by new strategies deemed to better support the achievement of intervention and implementation outcomes. Furthermore, the conditions that are required for respective strategies to be effective, i.e., their parameters for success, should be described (2).

(4) *Co-design implementation protocol*:

The purpose of step 4 is to clearly document and detail decisions made in previous steps to ensure that stakeholders can integrate these in everyday operations. The evaluation team co-designs an implementation protocol outlining the updated intervention and implementation outcomes, theories of change, and implementation strategies together with stakeholders who have been involved in steps 1–3. This should include a timeline specifying when to put implementation strategies in place and when to expect changes in intervention and implementation outcomes, facilitating the systematic continuation of the ongoing implementation process. The protocol can be complemented by additional materials that describe the planned implementation strategies and their target groups in more detail. When preparing the implementation protocol, the evaluation team should account for documents describing the existing implementation process that might already be in use.

(5) *Co-design evaluation protocol*:

The purpose of step 5 is to co-develop an evaluation protocol that stakeholders can use to systematically monitor their continued implementation based on the revised strategies and outcomes. An important goal of this process is to ensure that stakeholders gain full ownership of this approach and can self-evaluate implementation outcomes whenever feasible. For the development of the evaluation protocol, the evaluation team and stakeholders discuss indicators for the attainment of outcomes as well as data sources and measurement instruments to assess these indicators. Already available data sources, such as internal monitoring systems, as well as additional implementation outcome measures should be considered, with a focus on identifying pragmatic, user-friendly instruments that are appropriate to use in the respective context. The implementation outcome repository developed at the Centre for Implementation Science, King's College London, provides a helpful resource for selecting these measures (21). The evaluation team and stakeholders also select an evaluation design with feasible measurement points for self-evaluation and/or suitable time points for external monitoring. Furthermore, and similar to the logic of plan-do-study-act cycles (22), the evaluation protocol can describe iterations of the five steps of Implementation Mapping allowing for a continuous improvement of implementation strategies.

## Mapping the Implementation of Swiss Colorectal Cancer Screening Programs—A Practice Example

The above approach will be applied in an ongoing study aimed at understanding the strategies used to implement multiple organized colorectal cancer (CRC) screening programs in Switzerland. About half of all Swiss cantons have established or are in the process of establishing organized CRC screening programs. These programs aim to improve early detection of colorectal cancer by disseminating easily understandable information about CRC screening, providing low-threshold access and affordable procedures, and using a centralized system to invite and track program participants (23). Yet, little is known about how and why these programs work. By working closely with program leaders and other stakeholders, we will work to identify concrete avenues for improving the implementation of organized CRC screening programs in Switzerland, thereby improving program performance and reducing preventable colorectal cancer-related mortality.

The five steps of Implementation Mapping will be employed in the following way: (1) Across programs, we will map the key stakeholders involved at different levels of program implementation. These will be interviewed, individually and in focus groups, to illicit information about their experience with barriers to the implementation of organized CRC screening programs and their perceptions of what is needed to better navigate these barriers. (2) Interviews and focus groups together with a review of program documentation will also be used to identify intervention and implementation outcomes that have been defined for the different cantonal programs. Moreover, performance objectives for different stakeholder groups who are involved in the implementation will be defined. (3) In a third step, we will illicit information from stakeholders to identify the strategies that are currently used to integrate and maintain organized CRC screening programs in routine health services in Switzerland. This will help to understand the rationale that lies behind the choice of different strategies and to identify the implicit or explicit theories of change that underlie different programs. One output from this phase will be a generic theory of change for the existing implementation of Swiss CRC screening programs. We will then use the literature—based on a systematic integrative review—to assess the degree to which currently used implementation strategies are suited to address shared barriers that exist across programs. The goal of this assessment is to identify gaps in or needs for further modification of existing implementation and to provide suggestions for how to adapt, replace, or expand existing and/or design additional implementation strategies, as well as their parameters for success. An integral part of this work will be regular member checks to enhance the implementability and usability of suggestions made. (4) We will detail the adaptation processes and codify novel implementation strategies in designated CRC screening implementation protocols and provide concrete examples of how to apply these approaches in practice settings. The aim is to support current and future program stakeholders in solving existing implementation problems and to better navigate common challenges in Swiss CRC screening program implementation. Program stakeholders will be invited to review and provide feedback on all protocols. (5) Protocols will also contain concrete suggestions for how to monitor and evaluate the use of implementation strategies together with their intended implementation outcomes.

## Discussion

When planning to refine the existing implementation of an empirically supported intervention, it is crucial to include stakeholders' experience and build on the knowledge and skills already gained through previous implementation efforts. The adapted Implementation Mapping framework presented here provides practical step-by-step guidance on how to evaluate existing implementation in a participatory, stakeholder-centered approach. At the core of this approach are the concrete—rather than hypothetical—barriers and facilitators that stakeholders experience when implementing interventions in specific settings. These settings often differ from the more ideal conditions of research projects in that financial or human resources may be more scarce, organizational climate less optimal, or stakeholder engagement more volatile. As such, Implementation Mapping of existing implementation represents a promising approach for building the knowledge base on real world implementation.

Applying the adapted Implementation Mapping approach is not without challenges. First, the approach may cause concerns among stakeholders about failed implementation efforts being exposed. For example, if a sub-optimal organizational climate is identified as a key barrier to implementation, pointing to implementation leadership building as a strategy, this may unsettle organizational leaders involved in the Implementation Mapping. It is therefore important to consider stakeholders' roles, responsibilities, and interests in the implementation process and to navigate these with great sensitivity (5). Second, it can be challenging to find a shared language that can be used by and with all stakeholders in an Implementation Mapping process. This is important for building a constructive work relationship (3), mutual understanding, and trust. Although collaboration and communication competences are seen as essential for leading successful implementation projects, these are rarely targeted by implementation science training (24) and more practical guidance is needed on how to create successful participatory implementation processes. Finally, both researchers and stakeholders may have limited resources for conducting retrospective Implementation Mapping. For researchers, it may be difficult to obtain funding for adapting implementation processes that are already in progress, and for stakeholders, who are invested in complex implementation efforts, it may be challenging to find the time needed for an Implementation Mapping process. Finding a good balance between following the steps in detail and using economic ways to do so can include using available documentations, for example, to collect as much information as possible before conducting stakeholder workshops. Moreover, qualitative data collections can be designed efficiently with the goal of reaching high “information power,” while working with small samples (25).

Nevertheless, employing Implementation Mapping to evaluate existing implementation offers several benefits. Merging stakeholders' setting expertise, especially regarding local change processes, with implementation science expertise can provide useful information for identifying and targeting implementation challenges. Implicit assumptions explaining choices of current implementation strategies can be made explicit and potential mechanisms of action of implementation strategies are documented. Assessing the current state of implementation outcomes can serve as a baseline for studying future changes in implementation outcomes resulting from refined implementation efforts, just as a retrospective overview of employed implementation strategies can serve as a helpful reference point for interpreting this baseline. In summary, evaluating existing implementation can generate valuable information for the improvement of ongoing implementation efforts, and an adapted Implementation Mapping methodology offers a tool to guide this process.

## Author Contributions

M-TS, BA, and LCl conceptualized the article. LCa and EN contributed to the theoretical discussion about stakeholder engagement and existing implementation with a literature review. M-TS wrote the first draft of the manuscript and BA included the practice example. BA, LCa, EN, and LCl provided feedback on the manuscript. LCl wrote the contribution to the field statement. All authors approve of the final version of the article.

## Funding

The University of Zurich provides support for open access publishing of this article. Funding for the study Improving organised colorectal cancer screening programmes in Switzerland: An implementation science study is provided by Swiss Cancer Research (HSR-5224-11-2020).

## Conflict of Interest

The authors declare that the research was conducted in the absence of any commercial or financial relationships that could be construed as a potential conflict of interest.

## Publisher's Note

All claims expressed in this article are solely those of the authors and do not necessarily represent those of their affiliated organizations, or those of the publisher, the editors and the reviewers. Any product that may be evaluated in this article, or claim that may be made by its manufacturer, is not guaranteed or endorsed by the publisher.
